# Identification of Novel Inhibitors of the Type I Interferon Induction Pathway Using Cell-Based High-Throughput Screening

**DOI:** 10.1177/1087057116656314

**Published:** 2016-06-29

**Authors:** Zoe O. Gage, Andri Vasou, David W. Gray, Richard E. Randall, Catherine S. Adamson

**Affiliations:** 1 School of Biology, University of St Andrews, St Andrews, Fife, UK; 2 Biomedical Sciences Research Complex (BSRC), University of St Andrews, St Andrews, Fife, UK; 3 Drug Discovery Unit, University of Dundee, Dundee, UK

**Keywords:** interferon, innate immunity, IRF3, NF-κB, high-throughput screen (HTS)

## Abstract

Production of type I interferon (IFN) is an essential component of the innate immune response against invading pathogens. However, its production must be tightly regulated to avoid harmful effects. Compounds that modulate the IFN response are potentially valuable for a variety of applications due to IFN’s beneficial and detrimental roles. We developed and executed a cell-based high-throughput screen (HTS) targeting components that participate in and/or regulate the IRF3 and nuclear factor (NF)–κB branches of the IFN induction pathway. The assay detects activation of the IFN induction pathway via an enhanced green fluorescent protein (eGFP) reporter gene under the control of the IFNβ promoter and was optimized, miniaturized, and demonstrated suitable for HTS as robust Z′ factor scores of >0.6 were consistently achieved. A diversity screening set of 15,667 small molecules was assayed and two novel hit compounds validated that specifically inhibit the IFN induction pathway. We demonstrate that one of these compounds acts at or upstream of IRF3 phosphorylation. A second cell-based assay to detect activation of the IFN signaling (Jak-Stat) pathway via an eGFP reporter gene under the control of an IFN-stimulated response element (ISRE) containing MxA promoter also performed well (robust Z′ factor >0.7) and may therefore be similarly used to identify small molecules that modulate the IFN signaling pathway.

## Introduction

Type I interferon (IFN) production is beneficial to the host organism due to its essential role in the innate immune response against invading pathogens, particularly viruses. Secreted type I IFNs (IFNα/β) bind to the IFNAR1/2 cell surface receptor, activating the IFN signaling pathway and expression of hundreds of IFN-stimulated genes (ISGs), many of which have antiviral activities.^1^ Type I IFNs also exert important immunomodulatory, antiproliferative, and antitumor activities. Regulation of type I IFN levels is critical, not only to optimize their beneficial activities but also to minimize the harmful effects caused by prolonged activation. Dysregulation of type I IFN production has been implicated in the pathogenesis of autoimmune and inflammatory diseases.^2,3^ More recently, an increasing number of type I interferonopathies have been identified, in which a genetic mutation results in detrimental upregulation of type I IFN expression.^3,4^ Chronic type I IFN signaling during persistent viral infections also results in immune dysfunction that can be detrimental to the host.^5^ Overall, type I IFN is beneficial to the host, although production must be tightly regulated to avoid harmful effects.

Induction of type I IFN is complex and has been extensively reviewed.^1,6,7^ Briefly, type I IFN production occurs via multiple distinct routes and is dependent on the specific pathogen, the type of cell infected, and the stage of infection. Upon infection, pathogen-associated molecular patterns (PAMPs) are recognized by a variety of host cell pattern recognition receptors (PRRs). Once stimulated by the appropriate PAMP, PRRs recruit an adaptor molecule that feeds into common downstream signaling pathways that result in the activation of key transcription factors (nuclear factor [NF]–κB and IRF3 or IRF7), which induce the expression of type I IFNs. A commonly used potent inducer of type I IFNs is the Sendai virus (SeV) Cantell strain, which is rich in defective interfering particles (DIs) that robustly activate the cytosolic PRR RIG-I.^8,9^ RIG-I recruits the adapter MAVS, which feeds into the IRF3 and NF-κB branches of the IFN induction pathway. During IRF3 activation, kinases TBK1 and IKKε phosphorylate IRF3, causing exposure of a nuclear localization signal to facilitate IRF3 nuclear translocation.^1^ During NF-κB activation, the IKK kinase complex activates IκB phosphorylation and degradation, allowing NF-κB nuclear migration.^1,7^ Once in the nucleus, activated IRF3 and NF-κB cooperate, along with other transcription factors, to trigger optimal type I IFN expression.^1,7^

The IFN induction pathway involves many other components, particularly regulatory factors,^10^ than those described above, and thus a wide variety of potential targets exists. Small molecules that modulate the IFN induction pathway would be useful as (1) chemical tools to further investigate the type I IFN induction pathway, (2) therapeutics to treat diseases caused by dysregulation of the type I IFN response,^3,4^ (3) treatment of viral diseases as either activators to exploit type I IFN antiviral properties^11^ or inhibitors to block the detrimental effects of immune dysfunction during persistent viral infections,^5^ and (4) biotechnology applications (e.g., we recently demonstrated the utility of IFN inhibitors in the production of interferon-sensitive viruses for a range of applications).^12^

In this study, we developed a cell-based screen to identify novel compounds that modulate the IFN induction pathway. The assay detects activation of the IFN induction pathway via expression of enhanced green fluorescent protein (eGFP) under the control of the IFNβ promoter.^12,13^ IFN induction is activated by SeV (Cantell) infection; hence, compounds targeting the IRF3 or NF-κB branches of the IFN induction pathway have the potential to be identified. We conducted a high-throughput screen (HTS) and identified two novel inhibitors that specifically inhibit the IFN induction pathway.

## Materials and Methods

### Cell Lines, Sendai Virus, and IFN

The A549 cell-line (ECACC, Salisbury, UK) and derivatives were maintained in Dulbecco’s modified Eagle’s medium with 10% (v/v) fetal bovine serum and L-glutamine (2 mM). A549 reporter cell line derivatives, A549/pr(IFNβ).GFP and A549/pr(ISRE).GFP, contain an eGFP gene under the control of the IFNβ promoter or the MxA promoter (containing IFN-stimulated response elements [ISREs]), respectively.^12,13^ SeV Cantell (4000 HA U/mL) was purchased from Charles River Laboratories (North Franklin, CT) and used at a 1:100 dilution unless otherwise stated. Purified IFNα (NHS, Dundee, UK) was used at 10^4^ U/mL.

### Inhibitors and Compound Library

Inhibitors of the IFN response, BX795,^14^ TPCA-1,^15^ and ruxolitinib (Rux)^16^ (Selleck Chemicals, Munich, Germany), were prepared as 10-mM stocks in DMSO. Actinomycin D (AMD) and cyclohexamide (CHX) (Sigma, Dorset, UK) were prepared as 10-mM stocks in DMSO and ethanol, respectively. The Small Diversity Set Compound Library (Dundee Drug Discovery Unit [DDU], University of Dundee, UK) was used for HTS and consists of 15,667 compounds at 10 mM in DMSO. Hit compounds StA-IFN-1 (4-(1-acetyl-1*H*-indol-3-yl)-5-methyl-2,4-dihydro-3*H*-pyrazol-3-one) (Chembridge, San Diego, CA), StA-IFN-2 (4-{[4-(thieno[3,2-*d*]pyrimidin-4-yl)-1,4-diazepan-1-yl]methyl}benzonitrile) (Enamine, Monmouth Jt., NJ), StA-IFN-4 (2-[(4,5-dichloro-6-oxo-1(6*H*)-pyridazinyl)methyl]-8-methyl-4*H*-pyrido[1,2-*a*]pyrimidin-4-one) (Enamine, Monmouth, NJ), and StA-IFN-5 (6-methyl-4-phenyl-*N*-(pyridin-4-yl)quinazolin-2-amine) (Mcule, Budapest, Hungary) were stored as 10-mM stocks in DMSO. All compounds were used at the indicated concentrations.

### Cell-Based IFN Induction and IFN Signaling Reporter Assays

The A549/pr(IFNβ).GFP and A549/pr(ISRE).GFP reporter cell lines have previously been used to monitor the IFN induction and IFN signaling pathways, respectively.^12^ Briefly, A549/pr(IFNβ).GFP cells were seeded (9 × 10^4^ cells/cm^2^) into 96-well plates and incubated overnight. A549/pr(IFNβ).GFP reporter cells were infected with SeV (40 HA U/mL) to induce the IFN induction pathway. Sixteen hours postinfection, cells were fixed with 5% (v/v) formaldehyde and washed prior to measuring eGFP expression using an Infinite M200 Pro (Tecan, Männedorf, Switzerland) plate reader at an excitation/emission of 488/518 nm. A549/pr(ISRE).GFP cells were similarly seeded and incubated with purified IFNα (10^4^ U/mL) to activate the IFN signaling pathway. Forty hours post-IFN, treatment cells were fixed and washed and eGFP expression monitored as above. When appropriate, cells were treated with compound 2 h prior to induction of the IFN induction or IFN signaling pathways. eGFP reporter gene expression was determined as either signal-to-background (S/B) ratio or percentage inhibition. S/B ratio was calculated using the GFP signal in raw fluorescent units (RFU) as

$${\mathrm{RFU}}_{\left(\mathrm{Activated}\right)}{/\mathrm{RFU}}_{\left(\mathrm{Unactivated}\right)}$$

where activated represents SeV-infected cells and unactivated is uninfected cells. Percentage inhibition was calculated using the RFU of each well normalized to a percentage effect (% effect) of the positive control, calculated as

$${\%\mathrm{Effect}=[(\mathrm{RFU}}_{\mathrm{Activated}}{-\mathrm{RFU}}_{\mathrm{Test}}{)/(\mathrm{RFU}}_{\mathrm{Activated}}{-\mathrm{RFU}}_{\mathrm{Unactivated}})]*100.$$

### HTS and Dose-Response Assays

A single-point HTS using the DDU Small Diversity Set library in a 384-well format was performed using the A549/pr(IFNβ).GFP reporter assay described above with the following variations. Cells were seeded (9 × 10^4^ cells/cm^2^) into 384-well plates and 30 µM of compound added using an Echo 550 liquid handler (Labcyte, Sunnyvale, CA) 2 h prior to SeV infection. An EnVision (PerkinElmer, Waltham, MA) plate reader was used to measure eGFP expression at an excitation/emission of 485/535 nm. HTS data output was analyzed by the RFU of each well normalized to a % effect of the controls, calculated as detailed above for percentage inhibition. HTS quality control criteria for the acceptance of an assay plate are as follows: S/B ratio ≥2%, coefficient of variation <8% ((σ/µ)*100), and robust Z′ ≥0.5.

Robust Z′ factor was calculated with the following formula:

$$1-\frac{\left(3*\left(1.4826*\mathrm{MAD}\left[0\%\mathrm{inhibition}\left(\mathrm{raw data}\right)\right]\right)\right)+\left(3*\left(1.4826*\mathrm{MAD}\left[100\%\mathrm{inhibition}\left(\mathrm{raw data}\right)\right]\right)\right)}{\mathrm{MEDIAN}\left[0\%\mathrm{inhibition}\left(\mathrm{raw data}\right)\right]-\mathrm{MEDIAN}\left[\mathrm{100}\%\mathrm{inhibition}\left(\mathrm{raw data}\right)\right]}.$$

with MAD = mean absolute deviation. Each statistical parameter was determined for each assay plate and the overall screen.

Ten-point dose-response assays were performed using the A549/pr(IFNβ).GFP and A549/pr(ISRE).GFP reporter assays and the Echo 550 liquid handler via a 10-point twofold serial dilution (50 to 0.1 µM) of hit compounds. For determination of potency, a four-parameter logistic fit of the following form was used:

$$y=A+(B-A)/(1+{\left((10^{C})/x\right)}^{D}),$$

where *A* = % inhibition at bottom, *B* = % inhibition at top, *C* = 50% effect concentration (IC_50_), *D* = slope, *x* = inhibitor concentration, and *y* = % inhibition. ActivityBase XE (IDBS, Alameda, CA) was used for all data processing, with utilization of SARgen (IDBS) software.

### Cell Viability Assays

The AlamarBlue (AB) reagent (Life Technologies, Carlsbad, CA) was used to assess the effect of test compounds on cell viability. Seeded A549 cells were treated with compounds using a 10-point twofold serial dilution (50 to 0.1 µM) and incubated for 48 h. AB was added to a final concentration of 10% (v/v) and incubated for 4 h and fluorescence measured at an excitation/emission of 545/590 nm. The percentage reduction in AB was calculated using the following controls: 0% reduced (DMEM + AB) and 100% reduced (Cells + DMEM + AB).

To assess compound effect on global cellular protein synthesis, cells were treated with compound for 24 and 48 h prior to labeling with [^35^S]Met/Cys pro-mix (PerkinElmer) for 1 h. To determine the effect of compound on SeV replication, cells were treated for 2 h followed by infection for 18 h prior to labeling as above. Whole-cell lysates were separated by sodium dodecyl sulfate polyacrylamide gel electrophoresis (SDS-PAGE) followed by isotope incorporation visualization and quantification using a FLA-5000 phosphoimager (FujiFilm, Tokyo, Japan) and Image Studio software (Li-Cor, Lincoln, NE).

### Quantitative Reverse-Transcriptase PCR Assay

A quantitative reverse-transcriptase (qRT)–PCR assay determined compound effect on IFNβ and MxA gene transcript levels upon stimulation of the IFN induction or IFN signaling pathways, respectively. A549 cells were treated with compound (10 µM) 2 h prior to either SeV infection (4 h) or IFNα treatment (16 h). Total cellular RNA was extracted using phenol-chloroform separation with Trizol. Total messenger RNA (mRNA) was reverse transcribed using RevertAid reverse transcriptase and oligo d(T) primers (ThermoFisher Scientific, Perth, UK). Resultant complementary DNA (cDNA) was used to qPCR amplify IFNβ (forward primer: GCTTCTCCACTACAGCTCTTTC; reverse primer: CAGTA TTCAAGCCTCCCATTCA; nucleotides 40–155), MxA (forward primer: GCCTGCTGACATTGGGTATAA; reverse primer: CCCTGAAATATGGGTGGTTCTC; nucleotides 570–931), or actin (forward primer: GGCACCACACCTTCTA CAAT; reverse primer CCTTAATGTCACGCACGATTTC; nucleotides 257–640) using MESA Blue Mastermix (Eurogentec, Liege, Belgium) and an Mx3005P real-time PCR thermocycler (Stratagene, San Diego, CA). A standard curve was generated (5-point 10-fold serial dilution of DNA of known concentration) to facilitate absolute quantification using MxPro software (Stratagene). Actin was used to normalize values with respect to cell number.

### Immunoblotting and Immunofluorescence Microscopy

Immunoblotting was used to detect phosphorylated IRF3 (pIRF3) and STAT1 (pSTAT1) in A549 cells following compound treatment (10 µM) and stimulation of the IFN induction or IFN signaling pathways, respectively. To detect pIRF3, cells were treated with compound 2 h prior to SeV infection. Three hours postinfection, cells were lysed and subjected to SDS-PAGE/Western blot, followed by immunodetection with rabbit anti-pIRF3 antibody (Cell Signaling, Danvers, MA) and goat anti-rabbit IRDye680 conjugated secondary antibody (Li-Cor). To detect pSTAT1, cells were treated with compound 2 h prior to incubation with purified IFNα. Fifteen minutes post-IFNα treatment, cell lysates were processed as above and pSTAT1 detected with goat anti-pSTAT1 antibody (Santa Cruz, Heidelberg, Germany) and donkey anti-goat horseradish peroxidase (HRP)–conjugated secondary antibody (Santa Cruz). Actin was detected using mouse anti-actin antibody (Sigma, Dorset, UK) and goat anti-mouse IRDye800 conjugated secondary antibody (Li-Cor). Bands were visualized via an Odyssey CLx near-infrared scanner (Li-Cor) or enhanced chemiluminescence.

Immunofluorescence microscopy was used to determine the effect of compounds on IRF3 nuclear translocation. To visualize IRF3 nuclear translocation, A549 cells were seeded onto coverslips, treated with compound, and infected with SeV. Three hours postinfection, cells were fixed, permeabilized, and incubated with rabbit anti-IRF3 antibody (Santa Cruz) followed by donkey anti-rabbit Texas Red conjugated secondary antibody (Abcam, Cambridge, UK) together with DAPI (Sigma). Images were collected on a Nikon Microphot-FXA microscope (Nikon, Surrey, UK) (40× magnification) and processed using ImageJ64 software (ImageJ, NIH, Bethesda, MD).

## Results

### Development, Optimization, and Miniaturization of the A549/pr(IFNβ).GFP Reporter Assay to a Format Suitable for HTS

The A549/pr(IFNβ).GFP reporter cell line provides a straightforward method to detect activation of the IFN induction pathway via eGFP expression under the control of the IFNβ promoter following stimulation by virus infection.^12,13^ We previously used this A549/pr(IFNβ).GFP reporter assay to demonstrate that existing IFN inhibitors block the IFN induction pathway.^12^ We propose here that the A549/pr(IFNβ).GFP reporter assay can be used to identify novel compounds that modulate the IFN induction pathway.

To adapt our assay to a format suitable for HTS, we embarked on a program of assay optimization to maximize the S/B ratio and minimize the timescale. Variable parameters were sequentially optimized (**Suppl. Fig. S1**), and the resultant assay achieved a maximum S/B ratio of 2.6 ± 0.06. We next evaluated the robustness of assay performance using robust Z′ factor^17^ alongside S/B ratio and coefficient of variation (CV) (**Suppl. Table S1**). In the first instance, a 96-well plate format using activated and nonactivated A549/pr(IFNβ).GFP reporter cells was tested. The assay was then miniaturized to a 384-well plate format and a small-scale pilot screen (three plates) was performed using the DDU Small Diversity Set compound library. Overall, we demonstrate that the assay is robust, as a robust Z′ factor of ≥0.66 ± 0.03 was consistently achieved. We further verified our A549/pr(IFNβ).GFP reporter assay by demonstrating that two IFN induction pathway inhibitors, BX795^14^ and TPCA-1,^15^ reduced eGFP expression in activated A549/pr(IFNβ).GFP reporter cells in a dose-dependent manner (**Suppl. Fig. S2**). Overall, this provides proof of principle that our assay is suitable for use in diversity screening to identify compounds that modulate the IFN induction pathway.

### Diversity Screening Using the A549/pr(IFNβ).GFP Reporter Assay to Identify Compounds That Modulate the IFN Induction Pathway

**Figure 1.**
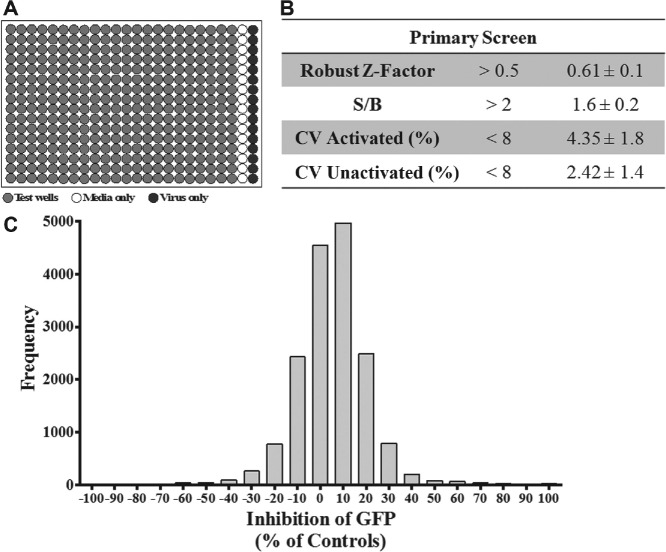
Single-point high-throughput screen (HTS) to identify compounds that modulate the interferon (IFN) induction pathway. A single-point HTS was performed using the A549/pr(IFNβ).GFP reporter assay and Drug Discovery Unit (DDU) small diversity set (*n* = 15,667 compounds). (**A**) A schematic of the 384-well plate layout; columns 1 to 22 contain a single test compound and Sendai virus (SeV)–infected cells, column 23 contains uninfected cells (maximum enhanced green fluorescent protein [eGFP] inhibition; 100% unactivated), and column 24 contains untreated SeV-infected cells (baseline eGFP inhibition; 0% activated). (**B**) Screen statistics compared with DDU preset quality control standards. (**C**) Screen output, represented as % effect of eGFP inhibition and plotted as a frequency distribution of all compounds tested. CV, coefficient of variation; S/B, signal-to-background ratio.

The A549/pr(IFNβ).GFP reporter assay was used to perform a single-point primary HTS using the DDU Small Diversity Set library (*n* = 15,667 compounds). Screening was performed in four batches of 12 × 384-well plates (**Fig. 1A**). The primary screen achieved a robust Z′ factor of 0.61 ± 0.1, which was comparable to that achieved during assay development, but the S/B ratio dropped to 1.6 ± 0.2 (**Fig. 1B**). Maintenance of the robust Z′ factor score indicates that the assay remained robust. A normal distribution of percentage effect was observed, centered at 0% to 10% inhibition, indicating that the screen performed as expected (**Fig. 1C**).

To be considered a hit, a compound was arbitrarily designated as having a percentage effect of greater than 50% or less than −50%, as well as 2 standard deviations above or below the test well average for the plate. Initially, these criteria resulted in 264 putative compounds. Duplicated compounds were withdrawn, followed by any compound that was shown to be toxic in previous DDU cell-based screens. This selection process reduced the number of hits to 245 (200 inhibited and 45 enhanced eGFP expression), yielding a primary screen hit rate of 1.3%.

**Figure 2.**
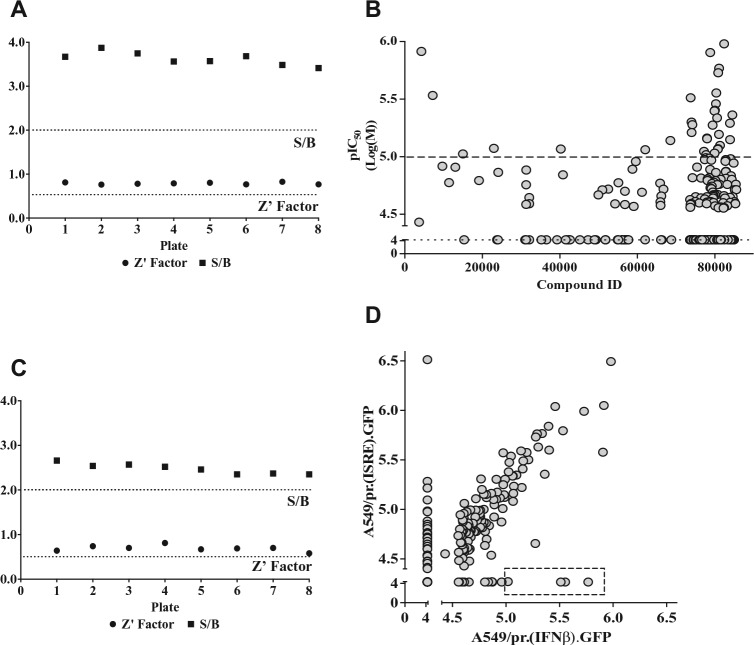
Secondary hit compound dose-response screening using A549/pr(IFNβ).GFP and A549/pr(ISRE).GFP reporter assays. Hit compound secondary screening using a 10-point dose-response curve (twofold dilution series from 50 to 0.1 µM) in the A549/pr(IFNβ).GFP and A549/pr(ISRE).GFP reporter assays. (**A**) Plate screen statistics for A549/pr(IFNβ).GFP reporter assays; Drug Discovery Unit (DDU) preset quality control standards (dotted lines). (**B**) pIC_50_ generated for each hit tested in the A549/pr(IFNβ).GFP reporter assay. Hits with a pIC_50_ of 4.3 (IC_50_ 50 µM) were deemed inactive (dotted line). Hits with an IC_50_ Log(M) ≥5 (IC_50_ ≤10 µM) were deemed favorable (dashed line). (**C**) Plate screen statistics for A549/pr(ISRE).GFP reporter assays; DDU preset quality control standards (dotted lines). (**D**) pIC_50_ generated for each hit tested in the A549/pr(IFNβ).GFP versus A549/pr(ISRE).GFP reporter assay. Boxed hits represent confirmed hit compounds that specifically inhibit the interferon (IFN) induction pathway and have a pIC_50_ ≥5 (IC_50_ ≤10 µM). Dose-response curves, pIC_50_ values, and statistics were generated using ActivityBase XE software (IDBS, Alameda, CA). S/B, signal-to-background ratio.

The 245 putative hits were taken forward to a secondary dose-response screen, which achieved a robust Z′ factor of 0.8 ± 0.02 and S/B ratio of 3.6 ± 0.1 (**Fig. 2A**). The dose-response curve for each compound was used to generate its IC_50_ value (**Fig. 2B**). Of the 245 hits tested, 109 were deemed inactive due to attaining an IC_50_ value greater than the highest concentration tested (50 µM) and were thus discarded. This eliminated all hits that enhanced eGFP expression. The remaining confirmed hits inhibited eGFP expression in a dose-dependent manner, and 41 were deemed the most favorable as they attained an IC_50_ value of ≤10 µM (**Fig. 2B**).

The original 200 putative inhibitors identified in the primary HTS were also subject to a specificity screen using a second reporter cell line, A549/pr(ISRE).GFP, which detects activation of the IFN signaling pathway via expression of eGFP under the control of the ISRE-containing MxA promoter following stimulation with purified IFNα.^12^ For each of the putative 200 hits, a dose-response screen using the A549/pr(ISRE).GFP reporter assay was performed, which achieved a robust Z′ factor of 0.7 ± 0.07 and S/B ratio of 2.5 ± 0.1 (**Fig. 2C**). The IC_50_ values for each compound generated in the A549/pr(IFNβ).GFP and A549/pr(ISRE).GFP reporter assays were plotted against each other (**Fig. 2D**). Of the 200 hits tested, 35 hits exhibited possible dual activity as they attained IC_50_ values of ≤10 µM in both assays. More important, 83 exhibited no activity in the A549/pr(ISRE).GFP reporter assay, strongly suggesting that their activity is specific to inhibition of the IFN induction pathway. Six of these IFN induction pathway-specific hits had an IC_50_ value of ≤10 µM and hence were considered confirmed hits.

### Validation of Hits That Specifically Inhibit the IFN Induction Pathway

**Figure 3.**
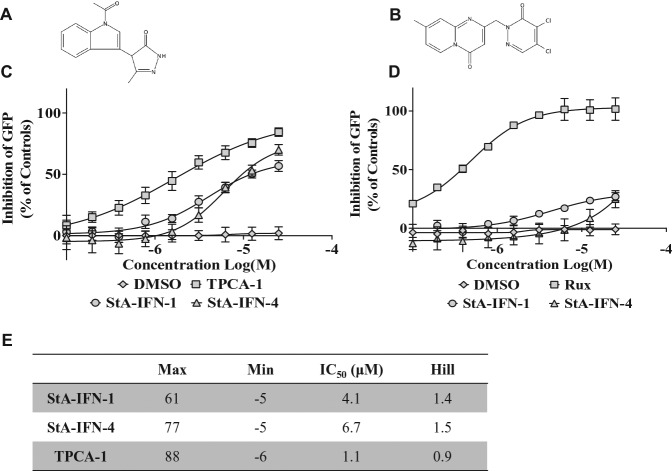
Potency and specificity of repurchased hit compounds StA-IFN-1 and StA-IFN-4. Repurchased hit compounds StA-IFN-1 (**A**) and StA-IFN-4 (**B**) were retested using a 9-point dose-response curve (twofold serial dilution series from 25 to 0.1 µM) using A549/pr(IFNβ).GFP reporter assay and TPCA-1 (**C**) or A549/pr(ISRE).GFP reporter assay and ruxolitinib (**D**). Data represent the mean of three independent experiments each conducted in triplicate; error bars indicate SD. (**E**) A549/pr(IFNβ).GFP reporter assay dose-response curve generated IC_50_, maximum, minimum, and Hill slope values using Prism 6 software (GraphPad Software, La Jolla, CA). GFP, green fluorescent protein.

The confirmed six hits were named StA-IFN-1, -2, -3, -4, -5, and -6. The hits were cherry picked from the compound library and analyzed by liquid chromatography–mass spectrometry (LC-MS) to confirm their identity. StA-IFN-3 and -6 failed LC-MS; hence, only StA-IFN-1, -2, -4, and -5 were repurchased and their activity and specificity confirmed by retesting in the two reporter assays. StA-IFN-2 and -5 were discontinued, as repurchased compound failed to exhibit pathway specificity (**Suppl. Fig. S3**). However, StA-IFN-1 and -4 (**Fig. 3A**,**B**) behaved as expected and were further examined by comparing their activity to the inhibitors TPCA-1, which inhibits the IFN induction pathway component IKKβ,^15^ and ruxolitinib, which inhibits IFN signaling pathway component Jak1^16^ (**Fig. 3C**,**D**). The IC_50_ values for StA-IFN-1 and -4 are in the µM range, which, along with maximum, minimum, and Hill slope values, are broadly comparable to TPCA-1 (**Fig. 3E**).

**Figure 4.**
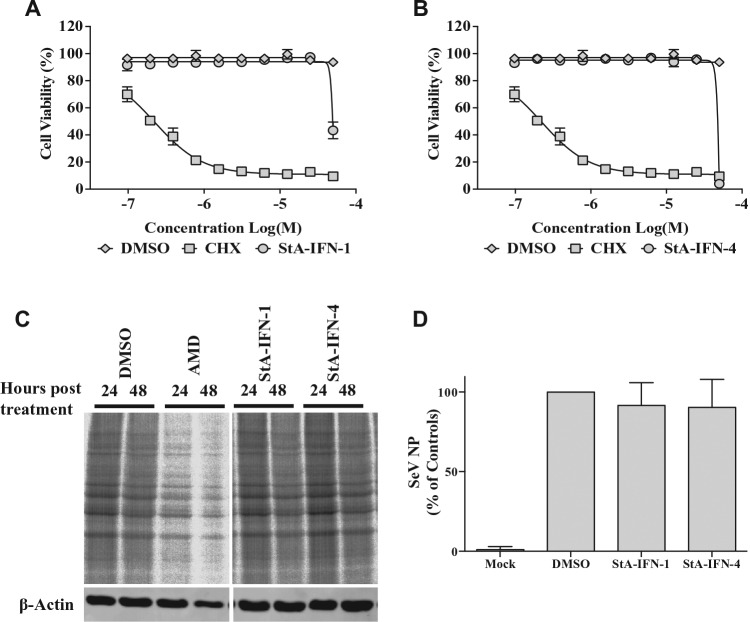
Elimination of off-target effects associated with StA-IFN-1 and StA-IFN-4. (**A, B**) Effect of StA-IFN-1 and StA-IFN-4 on A549 cell viability via an AlamarBlue assay; translational inhibitor, cyclohexamide (CHX), was used as a control. Data representative of three independent experiments, each conducted in quadruplicate, and error bars indicate SD. (**C**) Effect of StA-IFN-1 and StA-IFN-4 on A549 cellular protein synthesis; the transcriptional inhibitor, actinomycin D (AMD), was used as a control. A549 cells were treated with compound and then radiolabeled. Whole-cell lysates were separated by sodium dodecyl sulfate polyacrylamide gel electrophoresis (SDS-PAGE) and visualized by phosphoimager analysis. (**D**) Effect of StA-IFN-1 and StA-IFN-4 on Sendai virus (SeV) replication. A549 cells were treated with compound and infected with SeV. Eighteen hours postinfection, cells were radiolabeled. Whole-cell lysates were separated by SDS-PAGE and phosphoimager analysis used to quantitate SeV nucleoprotein (NP) levels. NP band intensity was quantified relative to a host cell protein and the DMSO control set at 100%. Data represent the mean of three independent experiments; error bars indicate SD.

To eliminate the possibility that StA-IFN-1 and -4 were false-positive hits, we demonstrated that these compounds did not (1) affect cell viability (**Fig. 4A**,**B**), (2) act as inhibitors of global protein synthesis (**Fig. 4C**), or (3) inhibit SeV infection and replication (**Fig. 4D**). Elimination of compounds with antiviral activity is particularly pertinent to our approach, as SeV infection is used to activate the IFN induction pathway, and a study using a similar IFNβ promoter-driven reporter assay has been used to identify flavivirus entry inhibitors.^18^ In contrast to StA-IFN-1 and -4, the five most promising dual-activity hits significantly reduced cellular protein synthesis (**Suppl. Fig. S4**); hence, these off-target compounds were not investigated further. Overall, the inhibitory effect of StA-IFN-1 and StA-IFN-4 is not due to off-target effects, and therefore these compounds are likely to specifically inhibit the IFN induction pathway.

**Figure 5.**
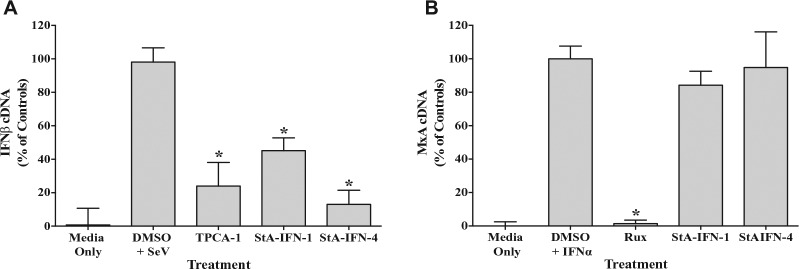
Effect of StA-IFN-1 and StA-IFN-4 on interferon β (IFNβ) and MxA messenger RNA (mRNA) levels. Effect of StA-IFN-1, StA-IFN-4, and TPCA-1 on IFNβ mRNA levels in A549 cells infected with Sendai virus (SeV) (**A**) or StA-IFN-1, StA-IFN-4 and Ruxolitinib on MxA mRNA levels in A549 cells activated with purified interferon α (IFNα) (**B**). Cells were treated with compound 2 h prior to activation. Three hours post-SeV infection and 18 h post-IFNα treatment, total cellular RNA was extracted and reverse transcribed. The resultant complementary DNA (cDNA) was used to quantitative PCR amplify either IFNβ or MxA sequences using appropriate primers. C_t_ values were subjected to absolute quantitation using a 6-point standard curve with DNA of known concentration and converted into % cDNA of controls. Data represent the mean of three independent experiments, each conducted in triplicate; error bars indicate SD. Statistical significance was assessed using the Student’s *t* test to compare compound treatment with the DMSO plus SeV or IFNα (*p < 0.0001). Rux, ruxolitinib.

Further validation was undertaken by examining compound effect on IFNβ and MxA mRNA levels (**Fig. 5**). As expected, TPCA-1, StA-IFN-1, and StA-IFN-4 significantly reduced IFNβ mRNA levels, directly indicating that these compounds inhibit the production of IFNβ. The inhibitory activity of StA-IFN-1 and -4 is specific to the IFN induction pathway, as these compounds had no effect on ISG MxA mRNA levels, whereas ruxolitinib, a known potent inhibitor of the IFN signaling pathway, significantly reduced MxA mRNA levels. These data confirm the inhibitory effect of StA-IFN-1 and -4 on the IFN induction pathway, independent of the reporter assay used during screening.

### StA-IFN-4 Inhibits the IFN Induction Pathway at or Upstream of IRF3 Phosphorylation

**Figure 6.**
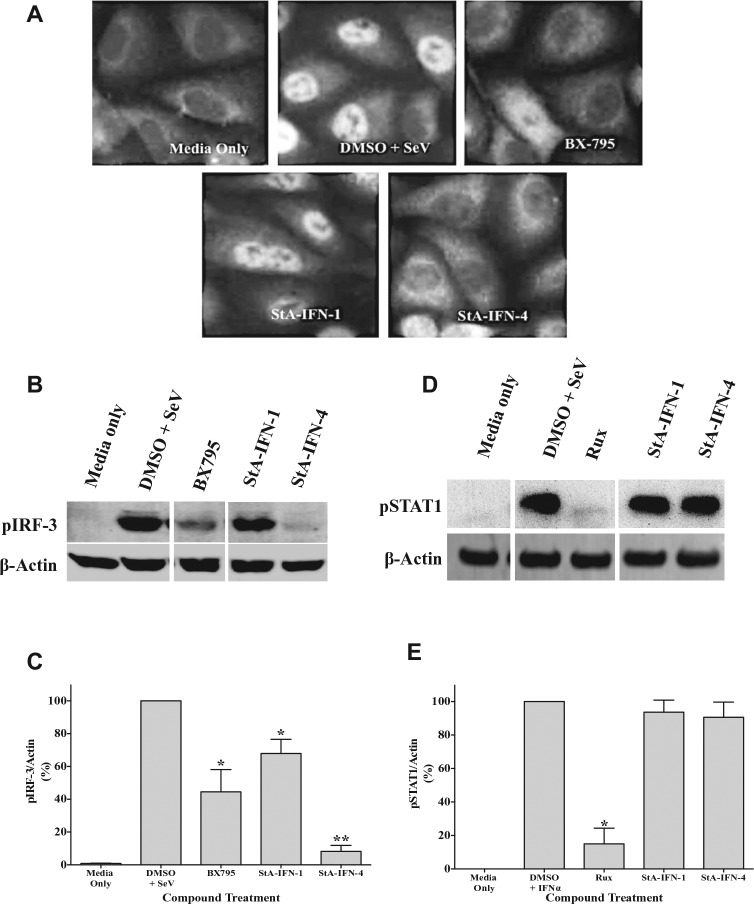
Effect of StA-IFN-1 and StA-IFN-4 on the IRF3 branch of the interferon (IFN) induction pathway. (**A**) Effect of StA-IFN-1, StA-IFN-4, and BX795 on IRF3 nuclear translocation in A549 cells infected with Sendai virus (SeV). Cells were treated with compound 2 h prior to activation. Three hours postinfection, cells were fixed, permeabilized, and probed with anti-pIRF3 antibody, followed by Texas Red–conjugated secondary antibody. Cells were visualized with a Nikon Microphot-FXA microscope (Nikon, Surrey, UK; 40× magnification). (**B, C**) Effect of StA-IFN-1, StA-IFN-4, and BX795 on pIRF3 levels in A549 cells infected with SeV. Cells were treated with compound 2 h prior to activation. Three hours postinfection, cells were lysed and subjected to sodium dodecyl sulfate polyacrylamide gel electrophoresis (SDS-PAGE)/Western blot, followed by detection with anti-pIRF3 or anti-actin antibody and IRDye680 or IRDye800-conjugated secondary antibody, respectively. Bands were visualized (**B**) and quantified as % pIRF3 relative to actin (**C**). (**D, E**) Effect of StA-IFN-1, StA-IFN-4, and ruxolitinib on pSTAT1 levels in A549 cells incubated with IFNα. Cells were treated with compound 2 h prior to activation. Fifteen minutes post-IFNα treatment, cells were lysed and subjected to SDS-PAGE/Western blot, followed by detection with anti-pSTAT1 or anti-actin antibody and horseradish peroxidase (HRP) or IRDye800-conjugated secondary antibody, respectively. Bands were visualized (**D**) and quantified as % pSTAT1 relative to actin (**E**). Statistical significance was assessed using the Student’s *t* test to compare compound treatment with the DMSO plus SeV or IFNα (*p < 0.05, **p < 0.0005). Mean values from three independent experiments are presented; error bars indicate SD.

We next instigated studies to determine the mechanism by which StA-IFN-1 and StA-IFN-4 target the IFN induction pathway. In the first instance, we investigated the IRF3 branch of the IFN induction pathway by determining if StA-IFN-1 and StA-IFN-4 block IRF3 nuclear translocation following IFN induction pathway activation via SeV infection (**Fig. 6A**). As expected, IRF3 is primarily cytoplasmic in noninfected cells but translocates to the nucleus upon SeV infection. Treatment with BX795, a known TBK1 inhibitor of the IRF3 branch of the IFN induction pathway,^14^ or StA-IFN-4 blocked IRF3 nuclear translocation as IRF3 remained predominantly cytoplasmic. In contrast, StA-IFN-1 did not affect IRF3 nuclear translocation.

We next demonstrated that StA-IFN-4 resulted in a dramatic reduction in phosphorylated IRF3 levels (**Fig. 6B**,**C**). StA-IFN-1 also caused a moderate reduction in pIRF3 levels. To eliminate the possibility that StA-IFN-4 is acting as a global phosphorylation inhibitor, we examined phosphorylated STAT1 (pSTAT1) levels. STAT1 is phosphorylated by the Jak1 kinase to activate the IFN signaling pathway.^1^ Following activation of the IFN signaling pathway, Jak1 inhibitor ruxolitinib significantly reduced the levels of pSTAT1, whereas StA-IFN-1 and -4 had no impact (**Fig. 6D**,**E**). Overall, these data suggest that StA-IFN-4 targets the IRF3 branch of the IFN induction pathway at or upstream of IRF3 phosphorylation.

## Discussion

Expression of an eGFP reporter gene under the control of the IFNβ promoter following virus infection was used to successfully develop and execute a cell-based HTS assay to identify novel inhibitors of the IFN induction pathway. The assay was miniaturized to a 384-well plate format and performed well as robust Z′ factors of >0.6 were consistently achieved. The assay has the capacity to target all components that participate in and/or regulate the IRF3 and NF-κB branches of the IFN induction pathway. This capacity to target multiple points in the signaling pathway provides the potential to identify novel compounds that target new or unknown pathway components.^19^ The NF-κB signaling pathway has been a major focus of drug discovery efforts, as this transcription factor regulates several important physiological processes, including the IFN induction pathway. As a consequence, hundreds of inhibitors targeting the NF-κB signaling pathway have been identified, some of which are available in the clinic.^20,21^ In contrast, relatively few compounds targeting components of the IRF3 branch of the IFN induction pathway have been reported. Efforts have primarily focused on TBK1/IKKε inhibitors, as these kinases play a key role in the IFN response and are also reported to be important in the development of certain human cancers.^14,22–26^

In this study, we identified and validated two novel compounds (StA-IFN-1 and StA-IFN-4) that specifically inhibit the IFN induction pathway. StA-IFN-4 is active against the IRF3 branch of the IFN induction pathway at or upstream of IRF3 phosphorylation as StA-IFN-4 dramatically reduced pIRF3 levels. In contrast, StA-IFN-1 only modestly reduced pIRF3 levels. Therefore, while StA-IFN-1 exhibits some activity at or before IRF3 phosphorylation, it may also act at another point in the activated pathway. While we have not eliminated the possibility that StA-IFN-1 acts downstream of IRF3 nuclear translocation, it is more likely that StA-IFN-1 also targets the NF-κB branch of the IFN induction pathway. Searches via SciFinder (Scifinder.cas.org) revealed several compounds with ~70% similarity to StA-IFN-1 that have been described as possible Tyr/Ser/Thr kinase inhibitors.^27^ Therefore, we speculate that StA-IFN-1 acts as a kinase inhibitor targeting the TBK1/IKKε and/or IKKα/IKKβ kinase complexes required for the IRF3 and NF-κB pathway branches, respectively. In contrast, StA-IFN-4 appears to be more novel; 85 molecules shared ≥70% similarity with StA-IFN-4, but only four publications/patents are associated with these molecules, none of which contained any indication relevant to the IFN response. In common with other phenotypic screens, further target deconvolution is required to determine the molecular target and mechanism of action of StA-IFN-1 and StA-IFN-4. Target deconvolution can be a substantial and lengthy undertaking, and its success may depend on a medicinal chemistry campaign to increase compound potency and is thus beyond the scope of this article.

Our assay also has the potential to identify compounds that enhance IFN production, and such compounds would be useful to exploit the antiviral properties of type I IFN.^11^ Unfortunately, the screen conducted in this study did not yield any validated hit compounds that enhanced IFN production. Eighteen compounds with IFN-inducing properties have previously been identified in an HTS of 94,398 small molecules.^11^ This work used 293T cells with a firefly luciferase reporter gene under the control of the IFNβ promoter, and like our assay, the IFN induction pathway was activated via SeV (Cantell) infection. However, the authors exclusively looked for IFN inducers, and so inhibitors of the IFN induction pathway were not reported. A notable difference between these IFNβ promoter assays using different reporter genes was the S/B ratio. Our eGFP-based assay typically achieved a low S/B ratio of 3, whereas the luciferase-based screen achieved a significantly higher S/B ratio of 11,934.61.^11^ While a high S/B ratio is preferable in assay development, the importance of this parameter is overridden by an excellent Z′ factor score, which better indicates assay quality as it takes into account both the assay signal window (S/B ratio) and data variability.^17^ Despite the relatively low S/B ratio attained in our assay, we consistently achieved a robust Z′ factor of >0.6, which indicated an excellent assay suitable for HTS. In addition, we choose to use an eGFP reporter gene because luciferase-based reporter gene assays are prone to compound interference.^28^ Nevertheless, the success of these screens validates this HTS approach as a strategy to discover small molecules that modulate the IFN response. In addition, we propose that the validation of our A549/pr(IFNβ).GFP and A549/pr(ISRE).GFP reporter cell lines permits them to be used as a platform to target viral IFN antagonists to discover novel antiviral compounds.

## Supplementary Material

Supplementary material

## References

[1] Randall R. E. Goodbourn S. Interferons and Viruses: An Interplay between Induction, Signalling, Antiviral Responses and Virus Countermeasures. J. Gen. Virol. 2008, 89(Pt 1), 1–47. 18089727 10.1099/vir.0.83391-0

[2] Lopez de Padilla C. M. Niewold T. B. The Type I Interferons: Basic Concepts and Clinical Relevance in Immune-Mediated Inflammatory Diseases. Gene 2016, 576(Pt 1), 14–21. 26410416 10.1016/j.gene.2015.09.058 PMC4666791

[3] Junt T. Barchet W. Translating Nucleic Acid–Sensing Pathways into Therapies. Nat. Rev. Immunol. 2015, 15, 529–544. 26292638 10.1038/nri3875

[4] Crow Y. J. Type I Interferonopathies: Mendelian Type I Interferon Up-Regulation. Curr. Opin. Immunol. 2015, 32, 7–12. 25463593 10.1016/j.coi.2014.10.005

[5] Snell L. M. Brooks D. G. New Insights into Type I Interferon and the Immunopathogenesis of Persistent Viral Infections. Curr. Opin. Immunol. 2015, 34, 91–98. 25771184 10.1016/j.coi.2015.03.002 PMC4444376

[6] Dempsey A. Bowie A. G. Innate Immune Recognition of DNA: A Recent History. Virology 2015, 479–480, 146–152. 10.1016/j.virol.2015.03.013 PMC4424081 25816762

[7] Schmitz M. L. Kracht M. Saul V. V. The Intricate Interplay between RNA Viruses and NF-kappaB. Biochim. Biophys. Acta 2014, 1843, 2754–2764. 25116307 10.1016/j.bbamcr.2014.08.004 PMC7114235

[8] Strahle L. Garcin D. Kolakofsky D. Sendai Virus Defective-Interfering Genomes and the Activation of Interferon-Beta. Virology 2006, 351, 101–111. 16631220 10.1016/j.virol.2006.03.022

[9] Baum A. Sachidanandam R. Garcia-Sastre A. Preference of RIG-I for Short Viral RNA Molecules in Infected Cells Revealed by Next-Generation Sequencing. Proc. Natl. Acad. Sci. U. S. A. 2010, 107, 16303–16308. 20805493 10.1073/pnas.1005077107 PMC2941304

[10] Chiang J. J. Davis M. E. Gack M. U. Regulation of RIG-I-Like Receptor Signaling by Host and Viral Proteins. Cytokine Growth Factor Rev. 2014, 25, 491–505. 25023063 10.1016/j.cytogfr.2014.06.005 PMC7108356

[11] Martinez-Gil L. Ayllon J. Ortigoza M. B. Identification of Small Molecules with type I Interferon Inducing Properties by High-Throughput Screening. PLoS One 2012, 7, e49049. 23145065 10.1371/journal.pone.0049049 PMC3492183

[12] Stewart C. E. Randall R. E. Adamson C. S. Inhibitors of the Interferon Response Enhance Virus Replication In Vitro. PLoS One 2014, 9, e112014. 25390891 10.1371/journal.pone.0112014 PMC4229124

[13] Chen S. Short J. A. Young D. F. Heterocellular Induction of Interferon by Negative-Sense RNA Viruses. Virology 2010, 407, 247–255. 20833406 10.1016/j.virol.2010.08.008 PMC2963793

[14] Clark K. Plater L. Peggie M. Use of the Pharmacological Inhibitor BX795 to Study the Regulation and Physiological Roles of TBK1 and IkappaB Kinase Epsilon: A Distinct Upstream Kinase Mediates Ser-172 Phosphorylation and Activation. J. Biol. Chem. 2009, 284, 14136–14146. 19307177 10.1074/jbc.M109.000414 PMC2682862

[15] Podolin P. L. Callahan J. F. Bolognese B. J. Attenuation of Murine Collagen-Induced Arthritis by a Novel, Potent, Selective Small Molecule Inhibitor of IkappaB Kinase 2, TPCA-1 (2-[(aminocarbonyl)amino]-5-(4-fluorophenyl)-3-thiophenecarboxamide), Occurs via Reduction of Proinflam-matory Cytokines and Antigen-Induced T Cell Proliferation. J. Pharmacol. Exp. Ther. 2005, 312 (1), 373–381. 15316093 10.1124/jpet.104.074484

[16] Quintas-Cardama A. Vaddi K. Liu P. Preclinical Characterization of the Selective JAK1/2 Inhibitor INCB018424: Therapeutic Implications for the Treatment of Myeloproliferative Neoplasms. Blood 2010, 115, 3109–3117. 20130243 10.1182/blood-2009-04-214957 PMC3953826

[17] Zhang J. H. Chung T. D. Oldenburg K. R. A Simple Statistical Parameter for Use in Evaluation and Validation of High Throughput Screening Assays. J. Biomol. Screen. 1999, 4, 67–73. 10838414 10.1177/108705719900400206

[18] Guo F. Zhao X. Gill T. An Interferon-Beta Promoter Reporter Assay for High Throughput Identification of Compounds against Multiple RNA Viruses. Antiviral Res. 2014, 107, 56–65. 24792753 10.1016/j.antiviral.2014.04.010 PMC4143146

[19] Zheng W. Thorne N. McKew J. C. Phenotypic Screens as a Renewed Approach for Drug Discovery. Drug Discov. Today 2013, 18, 1067–1073. 23850704 10.1016/j.drudis.2013.07.001 PMC4531371

[20] Gilmore T. D. Herscovitch M. Inhibitors of NF-kappaB Signaling: 785 and Counting. Oncogene 2006, 25, 6887–6899. 17072334 10.1038/sj.onc.1209982

[21] Herrington F. D. Carmody R. J. Goodyear C. S. Modulation of NF-kappaB Signaling as a Therapeutic Target in Autoimmunity. J. Biomol. Screen. 2016, 21, 223–242. 26597958 10.1177/1087057115617456

[22] Yu T. Yang Y. Yin de Q. TBK1 Inhibitors: A Review of Patent Literature (2011–2014). Expert Opin. Ther. Pat. 2015, 25, 1385–1396. 26293650 10.1517/13543776.2015.1081168

[23] McIver E. G. Bryans J. Birchall K. Synthesis and Structure-Activity Relationships of a Novel Series of Pyrimidines as Potent Inhibitors of TBK1/IKKepsilon Kinases. Bioorg. Med. Chem. Lett. 2012, 22, 7169–7173. 23099093 10.1016/j.bmcl.2012.09.063

[24] Johannes J. W. Chuaqui C. Cowen S. Discovery of 6-Aryl-Azabenzimidazoles That Inhibit the TBK1/IKK-Epsilon Kinases. Bioorg. Med. Chem. Lett. 2014, 24, 1138–1143. 24462666 10.1016/j.bmcl.2013.12.123

[25] Li J. Huang J. Jeong J. H. Selective TBK1/IKKi Dual Inhibitors with Anticancer Potency. Int. J. Cancer 2014, 134, 1972–1980. 24150799 10.1002/ijc.28507 PMC3947486

[26] Richters A. Basu D. Engel J. Identification and Further Development of Potent TBK1 Inhibitors. ACS Chem. Biol. 2015, 10, 289–298. 25540906 10.1021/cb500908d

[27] Arnold L. Maria J. Berlanga C. 2-Pyrazolin-5-ones as Tyrosine Kinase Inhibitors 2001. Patent Number: PCT/US2000/020628

[28] Auld D. S. Southall N. T. Jadhav A. Characterization of Chemical Libraries for Luciferase Inhibitory Activity. J. Med. Chem. 2008, 51, 2372–2386. 18363348 10.1021/jm701302v

