# Supplementary material for: Identification of Novel Inhibitors of the Type I Interferon Induction Pathway Using Cell-Based High-Throughput Screening
Source: J Biomol Screen. 2016 Jun 29;21(9):978–88. doi: 10.1177/1087057116656314 (PMC5030734; doi:10.1177/1087057116656314)
Supplement: Supplementary material [file 10.1177_1087057116656314_supp.pdf]

Supplementary Material

Identification of Novel Inhibitors of the Type I Interferon Induction Pathway using Cell-based High Throughput Screening

Zoe O Gage[1,2], Andri Vasou[1,2], David W Gray[3], Richard E Randall[1,2] and Catherine S Adamson[1,2*]

[1] School of Biology, University of St Andrews, [2] Biomedical Sciences Research Complex (BSRC), University of St Andrews, [3] Drug Discovery Unit, University of Dundee. [*] Corresponding author

Corresponding author: Dr Catherine Adamson, School of Biology, Biomolecular Sciences Building, University of St Andrews North Haugh, St Andrews Fife, KY16 9ST, Scotland UK. **email:** csa21@st-andrews.ac.uk

Supplementary Table 1. Quality control and assay performance following optimization.

	QC Approval	96 well	384 well
**Robust Z'-Factor**	> 0.5	0.63 ± 0.07	0.66 ± 0.03
**S/B Ratio**	> 2	3.6 ± 0.38	3.12 ± 0.23
**CV Hi (%)**	< 8	7.23 ± 1.06	6.75 ± 0.6
**CV Lo (%)**	< 8	5.89 ± 0.02	3.08 ± 0.5

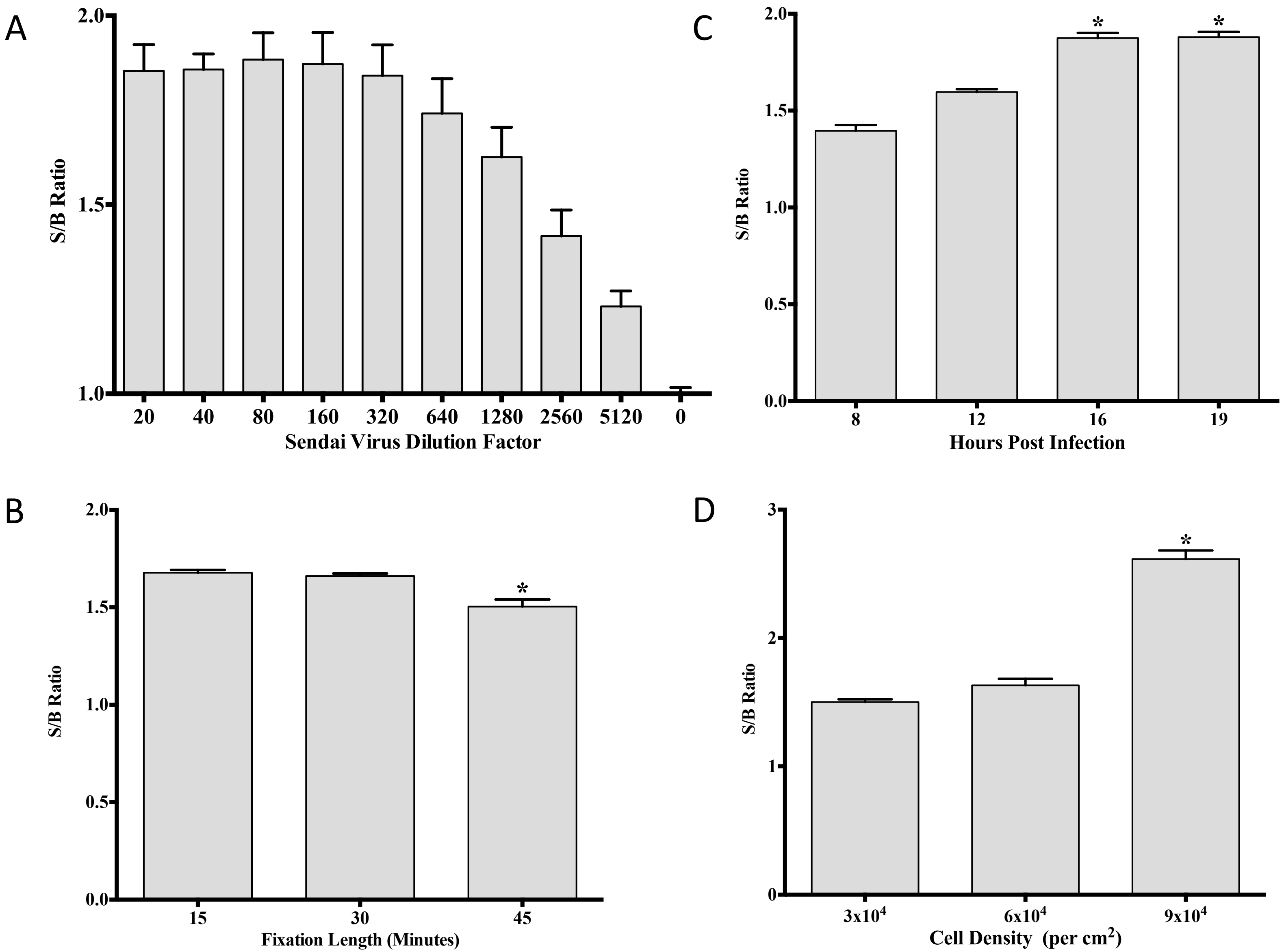
A
S/B Ratio
2.0
1.5
1.0
20
40
80
160
320
640
1280
2560
5120
0
Sendai Virus Dilution Factor
C
S/B Ratio
2.0
1.5
1.0
0.5
0.0
*
*
8
12
16
19
Hours Post Infection
B
S/B Ratio
2.0
1.5
1.0
0.5
0.0
*
15
30
45
Fixation Length (Minutes)
D
S/B Ratio
3
2
1
0
*
3x10^4
6x10^4
9x10^4
Cell Density (per cm^2)

Supplementary Fig.1 Optimization of the A549/pr(IFNβ).GFP reporter assay. Optimization of the A549/pr(IFNβ).GFP reporter assay in four successive steps; (A) SeV input, (B) length of SeV infection, (C) time of formaldehyde fixation and (D) cell seeding density. Level of eGFP reporter gene expression is presented as S/B ratio. The optimized assay utilizes cells seeded at 9 x 10^4 cells/cm^2, a 1:100 SeV dilution (40 HA units/ml), a 16 hours infection period followed by a 15-minute formaldehyde fixation to achieve the optimal S/B ratio in the minimum time. Data is representative of three independent experiments that were each conducted in quadruplicate; error bars indicate StDev. Statistical significance was assessed using the student's T-test to compare the S/B ratios achieved under differing assay conditions during each optimization step (* = $p<0.001$).

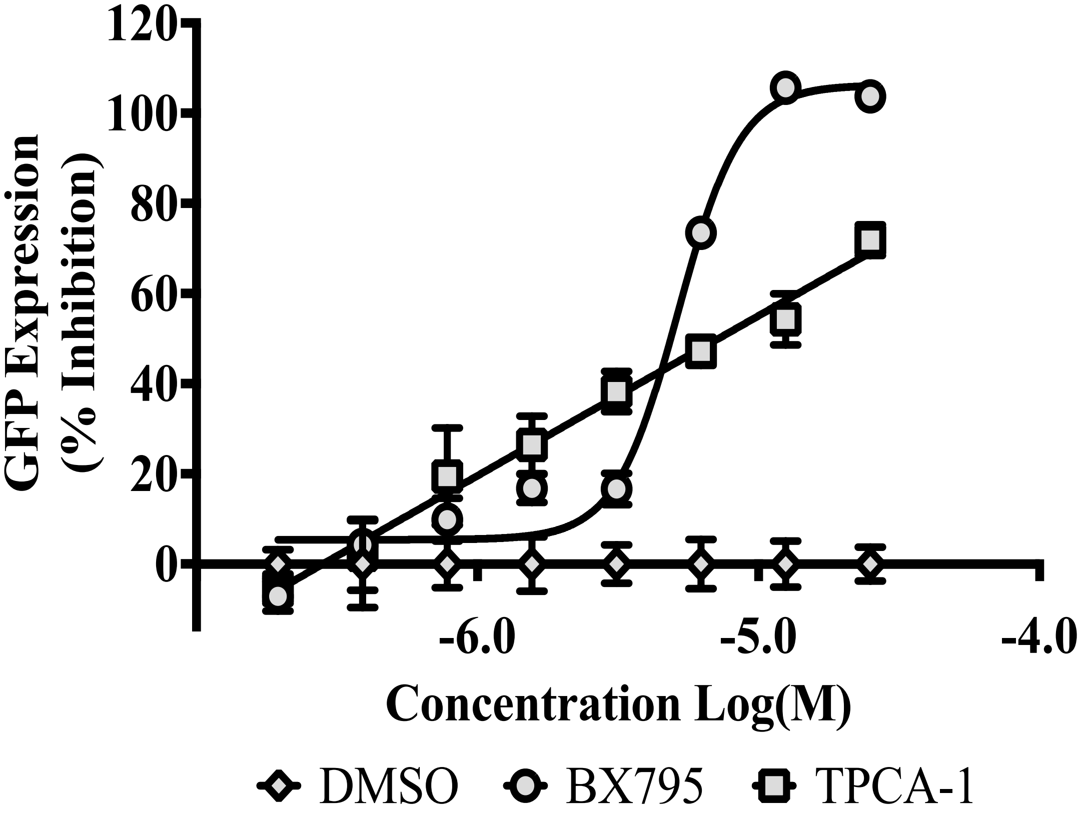

120
100
80
60
40
20
0
GFP Expression
(% Inhibition)
-6.0
-5.0
-4.0
Concentration Log(M)
DMSO
BX795
TPCA-1

Supplementary Fig.2. Verification of A549/pr(IFNβ).GFP reporter assay using IFN-induction pathway inhibitors. Small molecules reported to inhibit TBK1 (BX795) and IKKβ (TPCA-1) components of the IFN-induction pathway were utilized to verify the A549/pr(IFNβ).GFP reporter assay**.** The IFN-induction pathway and hence eGFP expression was activated by SeV infection and the effect of BX795 and TPCA-1 at various concentrations was measured by monitoring eGFP levels 16 hours post-infection. Data is presented as % effect of eGFP inhibition. Data is representative of three independent experiments that where each conducted in quadruplicate; error bars indicate StDev.

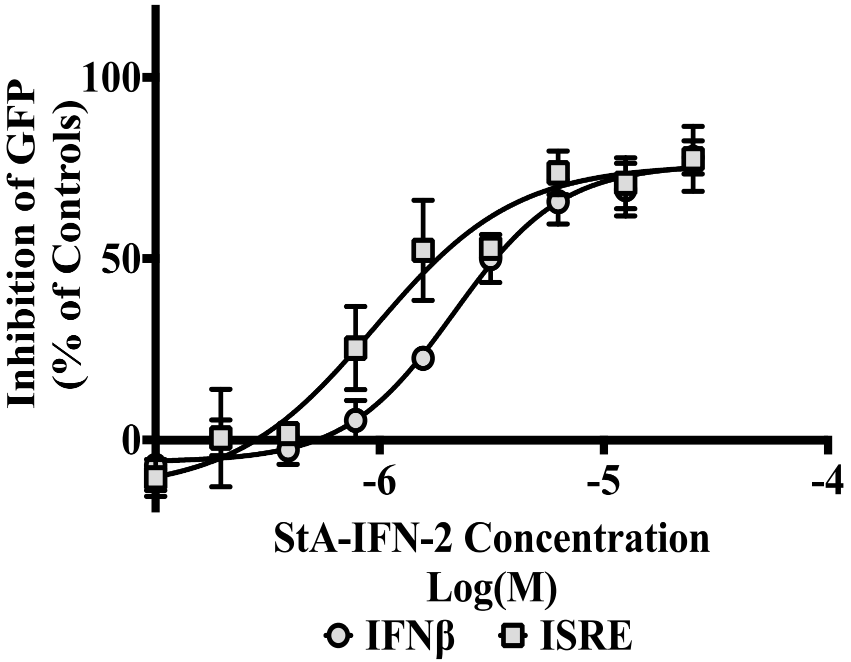

Inhibition of GFP
(% of Controls)
100
50
0
-6
-5
-4
StA-IFN-2 Concentration
Log(M)
IFNβ
ISRE

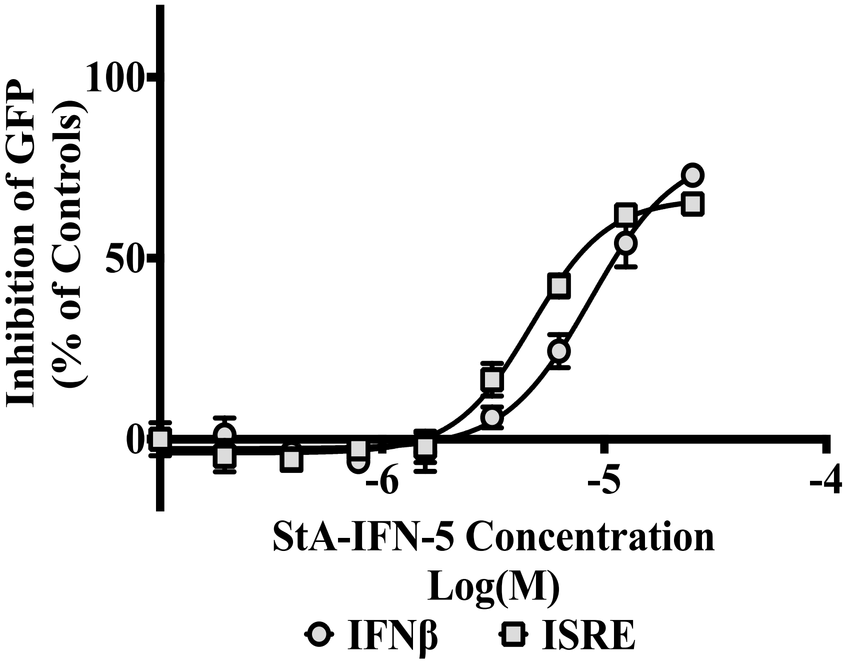

Inhibition of GFP
(% of Controls)
100
50
0
-6
-5
-4
StA-IFN-5 Concentration
Log(M)
IFNβ
ISRE

Supplementary Fig.3 Specificity of repurchased hit compounds StA-IFN-2 and StA-IFN-5. Repurchased hit compounds **(A)** StA-IFN-2 and **(B)** StA-IFN-5 were retested in using a 9-point dose-response curve via a 2-fold serial dilution series (25 to 0.1 μM) using A549/pr(IFNβ).GFP reporter assay and A549/pr(ISRE).GFP reporter assays. Data represents the mean of two independent experiments that were each conducted in triplicate; error bars indicate StDev.

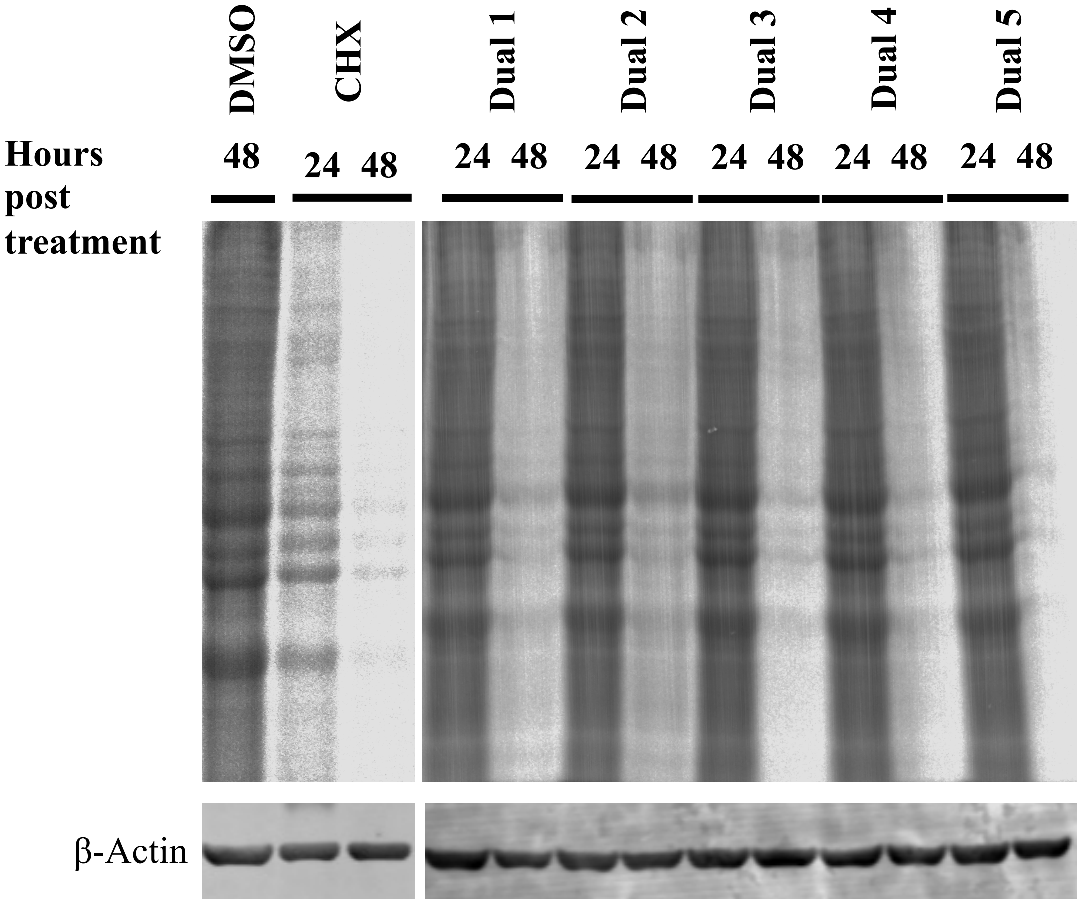
DMSO
CHX
Dual 1
Dual 2
Dual 3
Dual 4
Dual 5
Hours post treatment
48
24 48
24 48
24 48
24 48
24 48
24 48
β-Actin

Supplementary Fig.4 Off-target effect on cellular protein synthesis associated with hit compounds with dual activity. Effect of top five hit compounds that exhibited dual activity against the IFN-induction and IFN-signaling pathways on cellular protein synthesis. The transcriptional inhibitor, CHX was used as a control. Briefly, A549 cells were treated with compound for 24 or 48 hours and then radiolabelled. Whole cell lysates were separated by SDS/PAGE and visualized by phosphoimager analysis. Western blot and immunodetection was used to visualize β-actin.
